# Eicosapentaenoic Acid and Docosahexaenoic Acid as an Antimicrobial Agent in Orthopedics—An In Vitro Study About the Race for Surface

**DOI:** 10.3390/pathogens14010057

**Published:** 2025-01-10

**Authors:** Christopher Spiegel, Burak Ünalan, Andreas Kaserbacher, Rohit Arora, Débora C. Coraça-Huber

**Affiliations:** 1 Research Laboratory for Biofilms and Implant Associated Infections (BIOFILM LAB), University Hospital for Orthopaedics and Traumatology, Medical University of Innsbruck, Müllerstraße 44, 6020 Innsbruck, Austria; burak.uenalan@i-med.ac.at (B.Ü.); andreas.kaserbacher@student.i-med.ac.at (A.K.); debora.coraca-huber@i-med.ac.at (D.C.C.-H.); 2 Department of Orthopaedics and Traumatology, Medical University of Innsbruck, Anichstraße 35, 6020 Innsbruck, Austria; rohit.arora@tirol-kliniken.at

**Keywords:** biofilm, implant-related infections, PJI, omega-3 fatty acids, orthopedics, *Staphylococcus aureus*

## Abstract

Background: The burden of prosthetic joint infection in combination with antibiotic-resistant bacterial strains is a rising dilemma for patients experiencing total joint replacements. Around 0.8–2% of patients experience prosthetic joint infections, while up to 21% of patients are considered fatal cases after 5 years. *Staphylococcus aureus* is one of the main reasons for prosthetic joint infections. Its capability of forming biofilms and developing mechanisms against antibiotics is one of the most dangerous clinical topics being currently discussed. Previous studies have shown the promising results of omega-3 fatty acids as an antimicrobial agent against *Staphylococcus aureus*. Though an antimicrobial effect has been examined, the influence of polyunsaturated fatty acids on *Staphylococcus aureus* in the presence of human osteoblasts has not been reported yet. In this study, we aimed to investigate the influence of omega-3 fatty acids on the biofilm formation of *Staphylococcus aureus* ATCC 29213 in the presence of *hFOB 1.19* cells. The co-culture setup helped to examine the influence of omega-3 fatty acids on the race for surface to simulate prosthetic joint infections. Methods: In this study, we tested *Staphylococcus aureus* ATCC 29213 co-cultured with human fetal osteoblasts *hFOB 1.19* in the presence of sub-MIC and MIC concentrations of docosahexaenoic acid (1.25 mg/L, 2.5 mg/L) and eicosapentaenoic acid (0.15 mg/L, 0.3 mg/L) after 1, 6 and 24 h of incubation. After establishing the co-culture, cell culture and biofilm, we performed colony-forming unit counting and cell counting to examine cell survivability. In addition, we carried out scanning electron microscopy to study the race for surface behaviour of the cells. Results: We found a protective influence of omega-3 fatty acids on osteoblasts when present in co-culture with *Staphylococcus aureus* after 6 h of incubation. Omega-3 fatty acids increase the cell survival of osteoblasts after 6 h in a co-culture with bacteria and are able to influence the race for surface. In this study, the strain of *Staphylcoccus aureus* ATCC 29213 showed signs of growth inhibition within the first 6 h. Conclusions: Omega-3 fatty acids can be a valuable antimicrobial agent in terms of decreasing the risk of on-site infection during surgery. Omega-3 fatty acids were shown to decrease the bacterial load within the first 6 h of incubation and increase the survivability of osteoblasts.

## 1. Introduction

In the field of orthopedic surgery, bone and prosthetic joint infections (PJIs) remain a significant challenge. Infections follow approximately 0.8–2% of all prosthetic joint surgeries [1,2,3]. The mortality rate of PJIs is around 4% within the first year post-surgery, reaching up to 21% after 5 years post-surgery [4]. Moreover, more than 20–50% of open bone fractures lead to bone infections, and 25% of all clinical infections are related to device-associated infections [5]. The primary culprits behind medical device-related infections are coagulase-negative *Staphylococci* (CNS), *Staphylococcus aureus* strains and methicillin-resistant variants of *Staphylococcus aureus* (MRSA). These bacteria can cause both early (2–8 weeks) and late (3–36 months) implant infections [6]. Therapeutics for treating PJIs are limited. Antibiotic treatment combined with a revision surgery are the gold standard for treating PJIs. Each PJI revision surgery costs around USD 20.000–23.000 [7]. A significant challenge in treating these infections is bacteria’s ability to form biofilms. Staphylococci can form biofilms and develop methicillin resistance. Biofilm formation by *Staphylococcus aureus*, particularly methicillin-resistant *S. aureus* (MRSA), poses significant challenges in prosthetic joint infections (PJIs). Biofilms are structured communities of bacteria encased in a self-produced extracellular matrix, adhering to surfaces such as prosthetic materials [6,8,9,10,11]. This matrix not only impedes antibiotic penetration but also creates diffusion barriers that limit the access of antimicrobial agents and immune cells to the bacteria. These barriers contribute to the persistence of biofilm-encased bacteria by restricting nutrient flow and waste removal, forcing bacteria into a slow-growing or dormant state, which further reduces their susceptibility to antibiotics. MRSA’s resistance mechanisms, including the production of altered penicillin-binding proteins, further exacerbates treatment difficulties. Studies have shown that *S. aureus* is a leading cause of PJIs, with its biofilm-forming capability significantly contributing to infection persistence and recurrence [6]. Addressing these infections requires innovative strategies targeting both biofilm disruption and antibiotic resistance mechanisms to improve patient outcomes. Mechanisms such as bacterial cell linkage and the secretion of extracellular DNA (eDNA) stabilize staphylococcal biofilms [11]. Biofilm formation enables bacterial cells to endure harsh environmental conditions, including changes in pH, osmotic pressures, and increased antibiotic concentrations [12]. In the environment of implants, *Staphylococci* is able to destroy and nourish from the peri-implant tissue.

An important factor influencing infection outcomes is the “race for surface” theory. Traditionally, this theory suggests that host cells such as macrophages, fibroblasts, and platelets compete with bacteria to colonize the implant surface. Early host cell adhesion is thought to be key to preventing bacterial attachment and subsequent infection [13]. However, recent insights challenge this traditional notion, prompting a critical re-evaluation of the “race for surface” hypothesis. Several factors contribute to the colonization dynamics of surfaces, influencing whether bacteria or host cells dominate the implant colonization in the short term. These factors include the timing, quantity, and nature of surrounding cells, as well as the biomacromolecules in the local environment and the properties of the surface itself. Host cells tend to colonize the surface before bacteria. Depending on the bacterial strain, these host cells may either resist bacterial invasion or succumb to it [14].

To tackle already-infected implant sites, there is a needforf new antibacterial agents or substances with antibiotic-boosting activity. In previous studies, omega-3 fatty acids showed promising actions against *Staphylococci* biofilms. Coraça-Huber et al. were able to show that eicosapentaenoic acid (EPA) and docosahexaenoic acid (DHA) have antibacterial and anti-biofilm formation capabilities [15]. During several studies, DHA and EPA have shown MIC of 2.5 mg/L and 0.3 mg/L for *Staphylococcus aureus*. Also, herring oil and omega fatty acid compounds inhibit the biofilm formation of *Staphylococcus aureus* [16]. Omega-3 fatty acids show no increased biofilm formation when applied under minimal inhibition concentrations [17]. Omega-3 polyunsaturated fatty acid (PUFA) supplementation has also prompted an increase in osteoblastic differentiation levels [18].

This study aims to understand the influence of omega-3 fatty acids on osteoblasts and bacterial cells in a co-culture and the potential of omega-3 fatty acids as antimicrobial agents.

## 2. Materials and Methods

### 2.1. Substances

In this in vitro study, we utilized polyunsaturated fatty acids (PUFAs), docosahexaenoic acid (DHA; Cayman Chemical Company, Ann Arbor, MI, USA) and eicosapentaenoic acid (EPA; Cayman Chemical Company, Ann Arbor, MI, USA). We tested sub-minimal inhibitory concentrations (sub-MIC) and MIC of 1.25 mg/L and 2.5 mg/L DHA based on prior studies that determined the MIC for *Staphylococcus aureus* at 2.5 mg/L DHA by Coraca Huber et al. [15]. For EPA, we employed 0.15 mg/L and 0.3 mg/L. The PUFAs were diluted in tryptic soy broth with 1% glucose (TSB + 1% Glu; TSB, Merck KGaA, Darmstadt, Germany). Glucose is commonly used to improve the biofilm formation of *Staphylococcus aureus* [19,20]. Since all PUFAs were supplied dissolved in ethanol (EtOH), equal amounts of EtOH mixed with the media were used as solvent controls in the co-culture and osteoblast proliferation assays.

### 2.2. Cell Culture

Human fetal osteoblast cell line (*hFOB 1.19*) cells were pre-cultured through two passages until the achievement of 90% confluency. In the first subculture, 1 × 10^4^ osteoblasts were seeded in a 75 mL cell culture flask with 10 mL of Dulbecco’s Modified Eagle Medium (DMEM) and Nutrient Mixture F12 (Gibco, Life Technologies, Carlsbad, CA, USA), supplemented with 10% FCS and 1% Pen-Strep. The pre-culture was incubated at 34 °C with 95% humidity and 5% CO_2_ until 90% confluency was reached. Once the cells reached 90% confluency, a new passage was started with 1 × 10^4^ cells till 90% confluency was reached again. The media were changed every 48 h.

After reaching 90% confluency in the second passage, the cells were prepared for adhesion to the stainless-steel discs. The old media were removed and the cells were washed with 5 mL phosphate-free PBS. After the PBS was removed, 5 mL of TrypLE (Thermo Fisher Scientific, 168 Third Avenue, Waltham, MA, USA) was added. The cells were incubated for 5 min at 37 °C, and then trypsinization was stopped by adding 10 mL of DMEM F12 media. Next, 15 mL of the cell suspension was transferred to a 15 mL falcon tube and centrifuged at 200× *g* for 5 min. The supernatant was removed, and 2 mL of DMEM F12 media was added and mixed. The cells were then diluted to 1–2 × 10^4^ cells/mL, and 500 μL per well was added to a 96-well plate.

Before adding the cells, all steel discs were washed with sterile distilled water and autoclaved at 125 °C for 30 min. The discs were M2 discs made out of A2 steel with an outer diameter of 6 mm. After adding the cells to the steel samples in a 96-well plate, the setup was incubated for 1, 6 and 24 h at 37 °C with 95% humidity and 5% CO_2_. The temperature of 37 °C was chosen to reflect the physiological environment encountered in clinical scenarios. Osteoblast adhesion was assessed after 1, 6 and 24 h of culture. The used media were removed, each well was washed with 100 μL of PBS, and 100 μL of TrypLE was added. After a 5-min incubation at 37 °C, trypsinization was stopped by adding 100 μL of DMEM F12 media. The cells were extracted, and 200 µL of the cell suspension from each well was transferred to a 1.5 mL reaction tube. Finally, the cells were counted using a Trypan blue solution in a Neubauer-improved chamber.

### 2.3. Co-Culture

For the experiment, three colonies of *Staphylococcus aureus* ATCC 29213 were suspended in 2 mL of TSB +1% glucose media within a 15 mL centrifuge tube (VWR International, Radnor, PA, USA). The pre-cultures were incubated at 37 °C with shaking at 200 rpm overnight. Per PUFA, ethanol control and control triplicates of steel discs were established to examine different time points and parameters. Each concentration of PUFA, ethanol control and the control were diluted to reach the specific concentrations while diluting the bacterial down concentration to 10^2^ cells/mL. For the co-cultures, 10^4^ cells/mL *hFOB 1.19* cells were added to each well, finishing with a final volume of 200 μL per well. All 96-well plates were incubated for 1, 6 and 24 h at 37 °C with 95% humidity and 5% CO_2_. A temperature of 37 °C was chosen to reflect the physiological environment encountered in clinical scenarios.

Following the co-culture establishment, the remaining media were discarded. The steel samples were removed from the plate and washed with PBS to eliminate planktonic bacteria. The samples were then transferred to sterile vials, and 500 μL of TrypLE (Thermo Fisher Scientific, Waltham, MA, USA) was added to remove bacteria from the sample surfaces. Each vial was vortexed for 15 s before and after a 5-min incubation at 37 °C. To stop TrypLE activity, 500 μL of DMEM media was added, and the vial was vortexed again before proceeding with dilution. The samples were serially diluted from 10^−1^ to 10^−8^ in TSB +1% glucose, and dilutions from 10^−1^ after 1 h, 10^−3^ after 6 h and 10^−5^ after 24 h were plated in triplicate on Mueller–Hinton–Agar (MHA) plates. The plates were incubated for 48 h at 37 °C till colony-forming units (CFUs) were able to be counted.

The adhesion of osteoblasts was evaluated after 1, 6, and 24 h of culture. The culture media were removed, and each well was rinsed with 100 μL of PBS. Subsequently, 100 μL of TrypLE was added to each well. After a 5-min incubation at 37 °C, the trypsinization process was halted by adding 100 μL of DMEM F12 media. The cells were then collected, and 200 μL of the cell suspension from each well was transferred into a 1.5 mL reaction tube. Finally, the cells were counted using Trypan blue solution in a Neubauer-improved chamber.

### 2.4. Scanning Electron Microscopy

To examine biofilm formation and osteoblast proliferation on the steel samples, scanning electron microscopy (SEM) images were obtained using a JSM-6010LV microscope (JEOL GmbH, Freising, Germany). Following biofilm development and osteoblast proliferation, the steel samples were fixed overnight in 500 µL of 2.5% glutaraldehyde. After fixation, the samples underwent dehydration through a series of ethanol solutions at concentrations of 50%, 70%, 80%, and 99.9%, with each step lasting 5 min. The steel plates were then secured to aluminum pins using Leit-C adhesive strips (Göcke, Plano GmbH, Ahaus, Germany). Finally, the samples and pins were coated with gold for 40 s using an Agar Sputter Coater (Agar Scientific Ltd., Rotherham, UK).

### 2.5. Statistics

The analysis of all results was conducted using GraphPad Prism 10 (GraphPad Software, Inc., La Jolla, CA, USA). For a comprehensive statistical evaluation of CFU and viability data, a two-way analysis of variance (ANOVA) was applied, followed by post hoc analysis using Turkey’s multiple comparison tests. This additional step enabled detailed pairwise comparisons, allowing for the identification of significant differences between specific experimental conditions. This approach provided a nuanced assessment, taking into account the interplay between the various antibacterial agents and culture media utilized in the experiments. Further statistical data are displayed in Supplementary Tables S1–S7.

## 3. Results

### 3.1. Influence of Omega-3 Fatty Acids on Osteoblast Cell Culture

**Figure 1:**
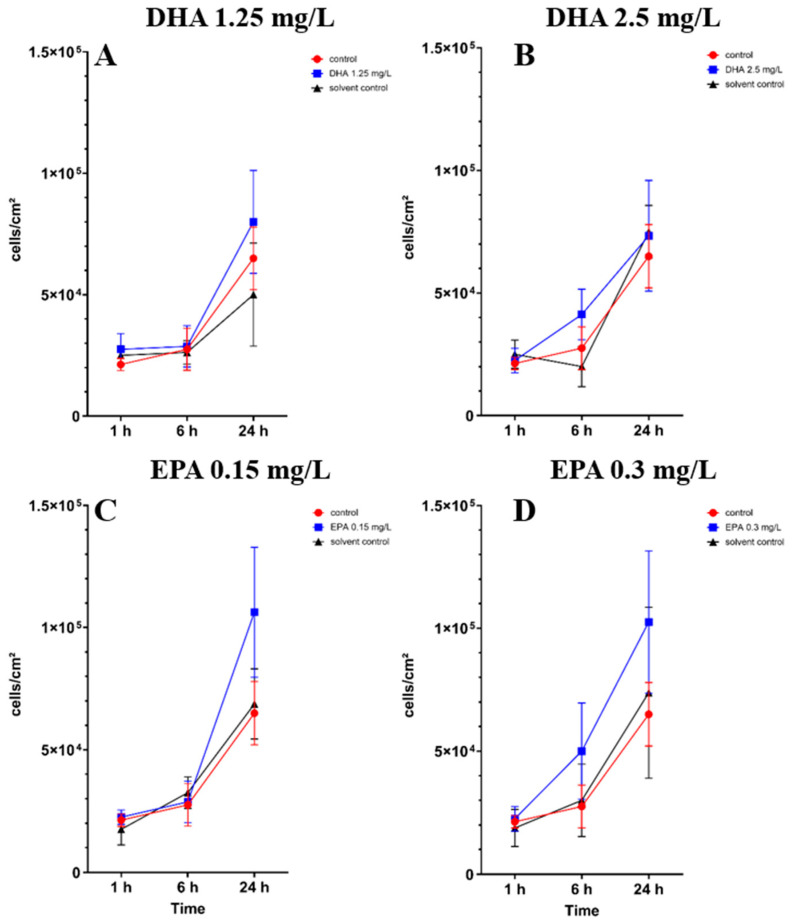
*hFOB 1.19* osteoblast cell count under the influence of PUFAs after 1, 6 and 24 h of incubation. The results are plotted against matching solvent controls and controls to understand the influence of PUFAs against ethanol. The differences between each nutrient medium and its glucose alternative were analyzed using a two-way ANOVA followed by Dunnett’s multiple comparison tests. The results are presented as mean values ± SD, derived from three independent experiments conducted in triplicate.

The PUFAs showed no significant decrease in adhesion of the osteoblasts in monoculture during the whole experiment (Table S1). In Figure 1A,C,D at 1.25 mg/L DHA, 0.15 mg/L EPA and 0.3 mg/L EPA, non-significant tendencies of an influence by omega-3 on osteoblast adhesion to the surface can be seen after 24 h of incubation. Overall, EPA seems to show a better separation between the solvent control and control towards the treated samples. In both concentrations, a tendency of influence of the PUFAs can be seen after 24 h of incubation. At 0.3 mg/L EPA (Figure 1D), after 6 h of incubation, a small separation of the treated group can be seen.

### 3.2. Influence of PUFAs on Co-Culture of Osteoblasts and Staphylococcus aureus

**Figure 2:**
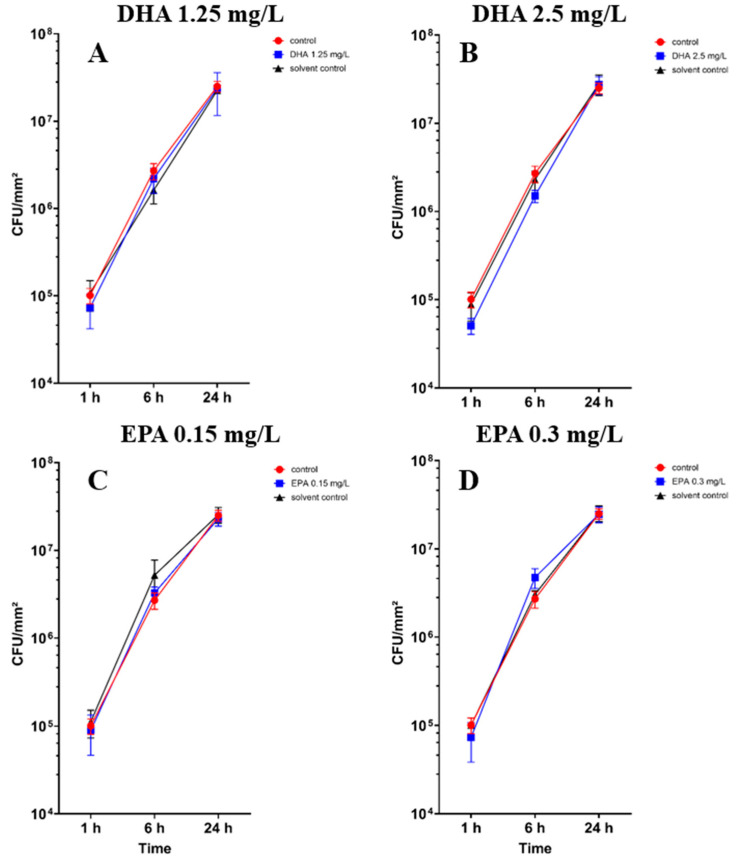
Colony-forming units of *Staphylococcus aureus* ATCC 29213 in co-culture with *hFOB 1.19* osteoblasts under the influence of PUFAs after 1, 6 and 24 h of incubation. The results are plotted against matching ethanol concentration and controls to understand the influence of PUFAs against ethanol. The differences between each nutrient medium and its glucose alternative were analyzed using a two-way ANOVA followed by Dunnett’s multiple comparison tests. The results are presented as mean values ± SD, derived from three independent experiments conducted in triplicate.

The colony-forming unit counts of *Staphylococcus aureus* ATCC 29213 in co-culture with *hFOB 1.19* cells are shown in Figure 2. There are no significant differences between the controls, solvent controls and the treated samples (Table S2). However, in Figure 2B, 2.5 mg/L DHA seems to show a tendency of separation towards the control and solvent control after 1 h and 6 h of incubation.

**Figure 3:**
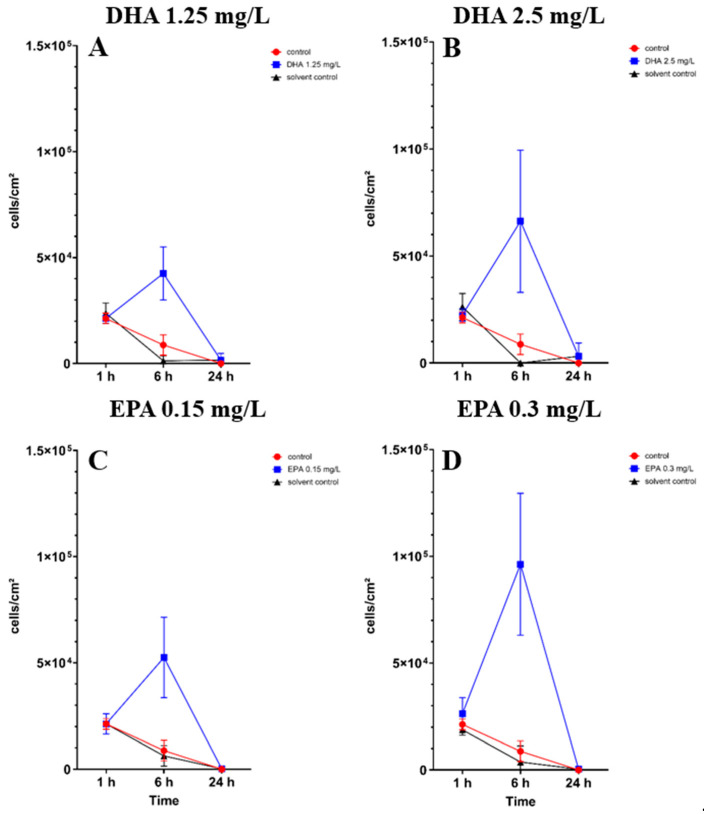
Osteoblast cell count in a co-culture with *Staphylococcus aureus* ATCC 29213 under the influence of PUFAs after 1, 6 and 24 h of incubation. The results show matching ethanol concentration and controls to understand the activity of PUFAs in the presence of ethanol. For a proper presentation, absolute cell counts are shown in the figures. The differences between each nutrient medium and its glucose alternative were analyzed using a two-way ANOVA followed by Dunnett’s multiple comparison tests. The results are presented as mean values ± SD, derived from three independent experiments conducted in triplicate.

In co-cultures with bacteria, the osteoblast cell count showed significant differences after 6 h of incubation in all concentrations of DHA and EPA (Tables S3–S7). At concentrations of 1.25 mg/L DHA, the cell count raised to 4.2 × 10^4^ cells/cm^2^ after 6 h of incubation in comparison to the solvent control with 1.2 × 10^3^ cells/cm^2^ and the control with 8.7 × 10^3^ cells/cm^2^ (Figure 3A). At 2.5 mg/L of DHA, the cell count increased to 6.6 × 10^4^ cells/cm^2^ after 6 h of incubation (Figure 3B). The solvent control hereby showed no cell count in co-culture after 6 h, whereas the control had a cell count of 8.7 × 10^3^ cells/cm^2^. EPA showed similar effects after 6 h of incubation. At 0.15 mg/L, the cell count was 5.2 × 10^4^ cells/cm^2^ while the solvent control showed a lower count of 6.2 × 10^3^ cells/cm^2^ (Figure 3C). At 0.3 mg/L, the cell count was at 9.6 × 10^4^ cells/cm^2^ and the solvent control was 3.7 × 10^3^ cells/cm^2^ (Figure 3D). After 24 h of incubation, unsignificant numbers of cells were found exclusively at 1.25 mg/L DHA and 2.5 mg/L DHA (Figure 3A,B). EPA, solvent controls and controls showed no viable cells after 24 h of incubation.

### 3.3. Scanning Electron Microscopy

**Figure 4:**
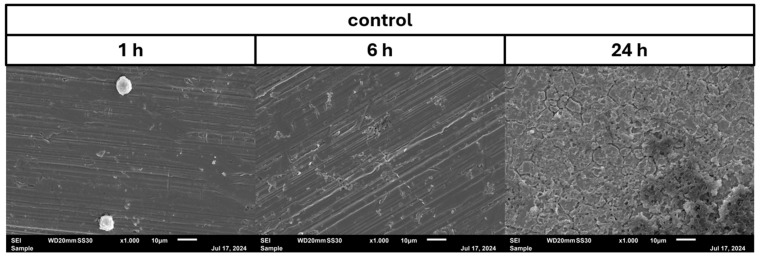
Control of osteoblast cell adhesion and growth after 1, 6 and 24 h in co-culture with *Staphylococcus aureus* ATCC 29213 at a PUFA concentration of 0 mg/L.

**Figure 5:**
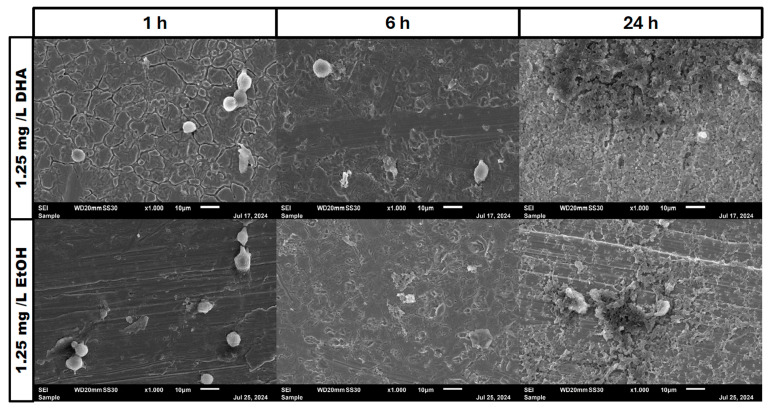
Osteoblast cell adhesion and growth after 1, 6 and 24 h in a co-culture with *Staphylococcus aureus* ATCC 29213 at a PUFA concentration of 1.25 mg/L DHA.

**Figure 6:**
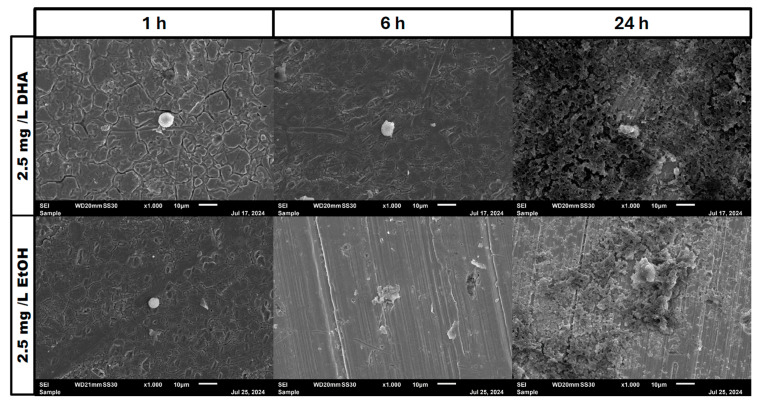
Osteoblast cell adhesion and growth after 1, 6 and 24 h in a co-culture with *Staphylococcus aureus* ATCC 29213 at a PUFA concentration of 2.5 mg/L DHA.

**Figure 7:**
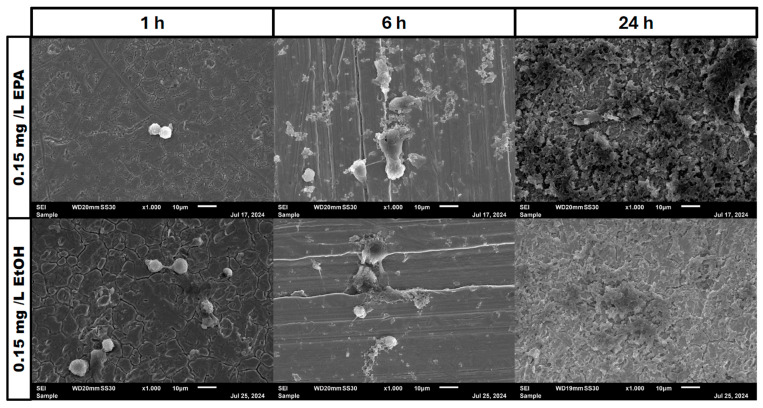
Osteoblast cell adhesion and growth after 1, 6 and 24 h in a co-culture with *Staphylococcus aureus* ATCC 29213 at a PUFA concentration of 0.15 mg/L EPA.

**Figure 8:**
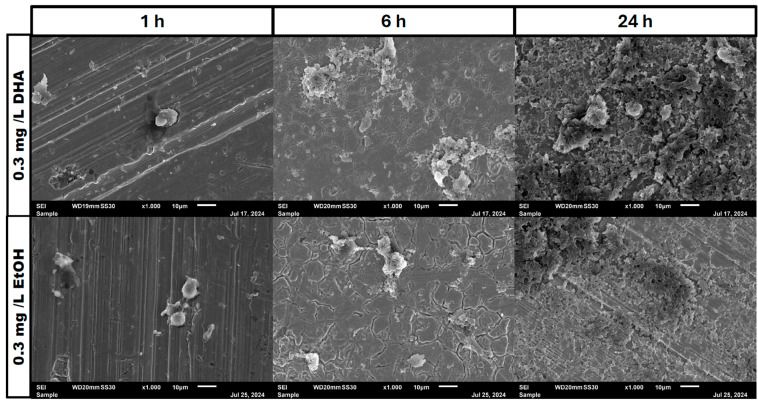
Osteoblast cell adhesion and growth after 1, 6 and 24 h in a co-culture with *Staphylococcus aureus* ATCC 29213 at a PUFA concentration of 0.3 mg/L EPA.

In the negative cells, scanning electron microscopy imaging showed no cells after 6 h of growth. After one hour, osteoblasts were adhered to the stainless-steel surface. After 24 h, a viable *Staphylococcus aureus* biofilm covered the surface of the samples (Figure 4). In the samples with 1.25 mg/L of DHA, more osteoblasts were visible after 1 h of incubation. While intact osteoblasts are present after 6 h of incubation, the ethanol control showed already destroyed osteoblasts after 6 h. After 24 h, a viable biofilm of *Staphylococcus aureus* ATCC 29213 was present (Figure 5). At a concentration of 2.5 mg/L DHA the osteoblasts were adhered after 1 h already to the surface. After 6 h cells without bacterial presence were visible. After 24 h of incubation no osteoblasts were visible under the biofilm of *Staphylococcus aureus*. The ethanol control of 2.5 mg/L DHA showed similar to 1.25 mg/L already destroyed osteoblasts after 6 h. After 24 h the biofilm has covered the whole surface of the sample (Figure 6). In the presence of 0.15 mg/L EPA, bacterial cells and osteoblasts were both visible after 6 h of incubation. Also, with the ethanol control, the same situation seen with the co-culture was present. After 24 h, no viable osteoblast cell was visible under the cover of bacterial biofilm. After 1 h, only osteoblasts were adhered to the stainless-steel sample surface (Figure 7). At 0.3 mg/L, the same situation as with the half concentration can be examined. After 1 h of incubation, the first osteoblasts adhered to the steel samples. After 6 h, both the osteoblasts and bacterial cells were present in the co-culture. After 24 h, a bacterial biofilm covered the steel sample surface (Figure 8). This can also be seen with the ethanol control for this concentration.

## 4. Discussion

The results of this study show that omega-3 fatty acids possess the ability to protect osteoblasts for a short term against *Staphylococcus aureus* ATCC 29213. In this study, we aimed to examine the influence of PUFAs on osteoblasts in a single culture and co-culture with *Staphylococcus aureus*. In previous studies, co-cultures of osteoblasts with *Staphylococcus aureus* have shown devastating effects on osteoblast survivability [21]. In the presence of antibiotic agents, the survivability was increased and continuous survival could have been established [22]. In the context of omega-3 fatty acids, previous studies have shown antimicrobial properties towards *Staphylococcus aureus* ATCC 25923 at concentrations of 2.5 mg/L for DHA and 0.3 mg/L for EPA [15]. Omega-3 fatty acids also have shown effects towards osteoblasts in terms of increasing differentiation and proliferation [14]. In this study, PUFAs show the ability to influence the race for surface. The osteoblasts at first adhere to the surface of all samples, whereas, after 6 h of incubation, the bacteria already engaged the osteoblasts.

The race for the surface is a concept published by Gristina et al. [13]. This concept claims that the first cell type to attach to a surface will be able to prevail and dominate the surface towards other cell types. As this is still a frequently referenced concept, it does not include the facts that *Staphylococcus aureus* has pathogenic virulence factors against human cells. These pathogenic virulence factors, like alpha-toxin or phenol-soluble modulins, can help to destroy cells to generate important nutrients for the bacterial cells and integrate into cells to survive [23].

In our study, we are able to show that the race for surface is non-existent. It is more like a race for survival for the osteoblasts, as *Staphylococcus aureus* is able to adhere and invade the osteoblast cell layer and overgrow the cells with a thick biofilm (Figure 4, Figure 5, Figure 6, Figure 7 and Figure 8). However, in the presence of omega-3 fatty acids, survivability is prolonged. After 6 h of incubation in the presence of all PUFAs and concentrations, significant numbers of osteoblasts were present. In a previous study, PUFAs have shown the capability to reduce bacterial counts but also influence the biofilm formation of *Staphylococcus aureus* [17]. Biofilm formation in *Staphylococcus aureus* is mainly driven by the icaADBC operon which expresses for polysaccharide intercellular adhesin (PIA) [6,9]. In a study by Spiegel et al., *Staphylococcus aureus* showed a decreased expression of icaADBC under the influence of PUFAs [17]. Another virulent factor which may come into play during this study are fibronectine binding proteins (FnBPBs) and clumping factors A and B [24,25,26,27,28,29]. PUFAs can increase cell membrane fluidity in human cells [29]. A change in cell membrane may alter the effective binding sites for *Staphylococcus aureus* and therefore decrease, in the short term, the binding and penetration sites for *Staphylococcus aureus*. In a clinical study, oral intake of Omega-3 fatty acids improved clinical outcomes against *Streptococcus pneumoniae* infections and had a protective effect on human cells [30]. The antioxidative effects of omega-3 fatty acids, which can also lead to reduce inflammation and lower infection rates, are frequently reported [31]. The bacteriostatic effect does not occur in a single culture of the bacteria [15]. In the co-culture, we were able to see a higher bacterial tolerance towards PUFAs. This is also an aspect to discuss for MIC testing of antibiotic substances, which are normally performed in single cultures and on agar plates [32]. The increase in CFU after 24 h of incubation in the co-culture can be explained due to oxidation effects or the consumption effect of the other cell types of omega-3 fatty acids [33]. Eukaryotic human cells are in need of omega-3 fatty acids to maintain the integrity of their cell membranes [34].

Omega-3 fatty acids have demonstrated promising anti-biofilm properties, making them a potential substance for combating bacterial biofilm formation and antibiotic resistance in PJIs. Integrating omega-3 into surgical infection management offers several possible application forms that must be carefully discussed to determine their effectiveness and feasibility in clinical practice. One potential method is a single intra-articular application directly on the site of infection during debridement or revision procedures. The local application in a single-stage procedure, similar to antibiotic irrigation, is common practice; however, it is expected that the omega-3 concentration on the wound site will drop exponentially within a few hours [35]. Another possibility is to incorporate PUFAs into PMMA bone cement in revisions. Although the heat resulting from bone-cementation processes can reach up to 70 °C, PUFAs like omega-3 fatty acids should not be influenced in their stability or oxidation [33]. This method would enable a slow, sustained release over time, helping to maintain therapeutic levels at the site of infection. A more innovative approach could involve the use of resorbable carriers or capsules containing omega-3 fatty acids. These capsules could be designed with varying dissolution times (e.g., 6, 12 or 24 h), providing a controlled, timed release of the agent. Lastly, omega-3 powder could be applied directly to the infection site or combined with antibiotic powders for a synergistic effect.

The study is limited to an in vitro design to show the effect of PUFAs on the concept of race for surface. Also, higher concentrations of omega-3 fatty acids may have shown more effective protection against *Staphylococcus aureus*. However, to understand the relation between bacteria and human cells, a scenario where bacteria are at an advantage seemed to be more realistic. Therefore, we also have chosen to incubate the co-culture setup at 37 °C in order to induce bacteria-favouring conditions. Additionally, only one bacterial strain has been used to prove the influence of the substances used. Although *Staphylococcus aureus* ATCC 29213 is a widely used bacterial strain in PJI studies, clinical strains and other species like *Staphylococcus epidermidis* or Gram-negative strains of *Enterobacteriaceae* or *Pseudomonas aeruginosa* could have been tested in this study. In previous studies with PUFAs, *Pseudomonas aeruginosa*, *Staphylococcus epidermidis* and *Staphylococcus aureus* have shown susceptibility towards DHA and EPA as antimicrobial agents [11]. The major limitation of this study is the in vitro approach of the experiments. An in vivo design with, for example, animal testing would give deeper insights into the applicability of PUFAs for treating PJIs. We suggest further investigations into the influence of PUFAs in combination with human cells and bacterial pathogens to fully understand the inhibition mechanisms and protective properties of PUFAs towards human cells.

## 5. Conclusions

PUFA supplementation can elongate the survivability of osteoblasts in a co-culture with *Staphylococcus aureus* ATCC 29213 cells. To use omega-3 PUFAs as a therapeutic agent, supplementation every 6 h locally to the site of infection should be considered. Also, higher concentrations of PUFAs should be considered and must be tested in further experiments.

## Data Availability

All data are presented in the publication.

## Supplementary Materials

The following supporting information can be downloaded at: www.mdpi.com/article/10.3390/pathogens14010057/s1, Table S1: *p*-values of the two-way ANOVA for the different time points and PUFAs used for osteoblast adhesion tests; Table S2: *p*-values of the two-way ANOVA for the different time points and PUFAs used of the colony forming unit counts of the co-culture; Table S3: *p*-values of the two-way ANOVA for the different time points and PUFAs used of the osteoblast cell counts of the co-culture; Table S4: Results of a Turkey’s multi-comparison test of the osteoblast cell counts of the co-culture with the control, solvent control and DHA 1.25 mg/L; Table S5: Results of a Turkey’s multi-comparison test of the osteoblast cell counts of the co-culture with the control, solvent control and DHA 2.5 mg/L; Table S6: Results of a Turkey’s multi-comparison test of the osteoblast cell counts of the co-culture with the control, solvent control and EPA 0.15 mg/L; Table S7: Results of a Turkey’s multi-comparison test of the osteoblast cell counts of the co-culture with the control, solvent control and EPA 0.3 mg/L.

## References

[1] Ahmed S.S., Begum F., Kayani B., Haddad F.S. (2019). Risk factors, diagnosis and management of prosthetic joint infection after total hip arthroplasty. Expert Rev. Med. Devices.

[2] Jin X., Luxan B.G., Hanly M., Pratt N.L., Harris I., de Steiger R., Graves S.E., Jorm L. (2022). Estimating incidence rates of periprosthetic joint infection after hip and knee arthroplasty for osteoarthritis using linked registry and administrative health data. Bone Jt. J..

[3] Tao Y., Luo Y., Hu H., Wang W., Zhao Y., Wang S., Zheng Q., Zhang T., Zhang G., Li J. (2024). Clinically applicable optimized periprosthetic joint infection diagnosis via AI based pathology. NPJ Digit. Med..

[4] Natsuhara K.M., Shelton T.J., Meehan J.P., Lum Z.C. (2019). Mortality During Total Hip Periprosthetic Joint Infection. J. Arthroplast..

[5] Okike K., Bhattacharyya T. (2006). Trends in the management of open fractures. A critical analysis. JBJS.

[6] Arciola C.R., Campoccia D., Montanaro L. (2018). Implant infections: Adhesion, biofilm formation and immune evasion. Nat. Rev. Microbiol..

[7] Kurtz S.M., Higgs G.B., Lau E., Iorio R.R., Courtney P.M., Parvizi J. (2022). Hospital Costs for Unsuccessful Two-Stage Revisions for Periprosthetic Joint Infection. J. Arthroplast..

[8] Arciola C.R., Baldassarri L., Montanaro L. (2002). In catheter infections by *Staphylococcus epidermidis* the intercellular adhesion (*ica*) locus is a molecular marker of the virulent slime-producing strains. J. Biomed. Mater. Res..

[9] Arciola C.R., Campoccia D., Ravaioli S., Montanaro L. (2015). Polysaccharide intercellular adhesin in biofilm: Structural and regulatory aspects. Front. Cell. Infect. Microbiol..

[10] Campoccia D., Baldassarri L., Pirini V., Ravaioli S., Montanaro L., Arciola C.R. (2008). Molecular epidemiology of *Staphylococcus aureus* from implant orthopaedic infections: Ribotypes, agr polymorphism, leukocidal toxins and antibiotic resistance. Biomaterials.

[11] Montanaro L., Poggi A., Visai L., Ravaioli S., Campoccia D., Speziale P., Arciola C.R. (2011). Extracellular DNA in Biofilms. Int. J. Artif. Organs.

[12] Rachid S., Cho S., Ohlsen K., Hacker J., Ziebuhr W. (2000). Induction of Staphylococcus epidermidis biofilm formation by environmental factors: The possible involvement of the alternative transcription factor sigB. Adv. Exp. Med. Biol..

[13] Gristina A.G., Naylor P., Myrvik Q. (1988). Infections from biomaterials and implants: A race for the surface. Med. Prog. Technol..

[14] Hickok N.J., Li B., Oral E., Zaat S.A.J., Armbruster D.A., Atkins G.J., Chen A.F., Coraça-Huber D.C., Dai T., Greenfield E.M. (2024). The 2023 Orthopedic Research Society’s international consensus meeting on musculoskeletal infection: Summary from the in vitro section. J. Orthop. Res..

[15] Coraça-Huber D.C., Steixner S., Wurm A., Nogler M. (2021). Antibacterial and Anti-Biofilm Activity of Omega-3 Polyunsaturated Fatty Acids against Periprosthetic Joint Infections-Isolated Multi-Drug Resistant Strains. Biomedicines.

[16] Kim Y.-G., Lee J.-H., Raorane C.J., Oh S.T., Park J.G., Lee J. (2018). Herring Oil and Omega Fatty Acids Inhibit *Staphylococcus aureus* Biofilm Formation and Virulence. Front. Microbiol..

[17] Spiegel C., Steixner S.J.M., Coraça-Huber D.C. (2022). Antibiofilm Activity of Omega-3 Fatty Acids and Its Influence on the Expression of Biofilm Formation Genes on *Staphylococcus aureus*. Antibiotics.

[18] Levental K.R., Surma M.A., Skinkle A.D., Lorent J.H., Zhou Y., Klose C., Chang J.T., Hancock J.F., Levental I. (2017). ω-3 polyunsaturated fatty acids direct differentiation of the membrane phenotype in mesenchymal stem cells to potentiate osteogenesis. Sci. Adv..

[19] Agarwal A., Jain A. (2013). Glucose & sodium chloride induced biofilm production & ica operon in clinical isolates of staphylococci. Indian J. Med. Res..

[20] Steixner S.J.M., Spiegel C., Dammerer D., Wurm A., Nogler M., Coraça-Huber D.C. (2021). Influence of Nutrient Media Compared to Human Synovial Fluid on the Antibiotic Susceptibility and Biofilm Gene Expression of Coagulase-Negative *Staphylococci* In Vitro. Antibiotics.

[21] Parente R., Possetti V., Schiavone M.L., Campodoni E., Menale C., Loppini M., Doni A., Bottazzi B., Mantovani A., Sandri M. (2021). 3D Cocultures of Osteoblasts and *Staphylococcus aureus* on Biomimetic Bone Scaffolds as a Tool to Investigate the Host–Pathogen Interface in Osteomyelitis. Pathogens.

[22] Stracquadanio S., Musso N., Costantino A., Lazzaro L.M., Stefani S., Bongiorno D. (2021). Internalization in Osteoblast Cells: Mechanisms, Interactions and Biochemical Processes. What Did We Learn from Experimental Models?. Pathogens.

[23] Cheung G.Y.C., Bae J.S., Otto M. (2021). Pathogenicity and virulence of. Virulence.

[24] Gongadze E., Kabaso D., Bauer S., Slivnik T., Schmuki P., Rienen U.V., Iglič A. (2011). Adhesion of osteoblasts to a nanorough titanium implant surface. Int. J. Nanomed..

[25] Houston P., Rowe S.E., Pozzi C., Waters E.M., O’Gara J.P. (2011). Essential Role for the Major Autolysin in the Fibronectin-Binding Protein-Mediated *Staphylococcus aureus* Biofilm Phenotype. Infect. Immun..

[26] Josse J., Laurent F., Diot A. (2017). Staphylococcal Adhesion and Host Cell Invasion: Fibronectin-Binding and Other Mechanisms. Front. Microbiol..

[27] Hawkins J., Kodali S., Matsuka Y.V., McNeil L.K., Mininni T., Scully I.L., Vernachio J.H., Severina E., Girgenti D., Jansen K.U. (2012). A Recombinant Clumping Factor A-Containing Vaccine Induces Functional Antibodies to *Staphylococcus aureus* That Are Not Observed after Natural Exposure. Clin. Vaccine Immunol..

[28] Foster T.J. (2016). The remarkably multifunctional fibronectin binding proteins of *Staphylococcus aureus*. Eur. J. Clin. Microbiol. Infect. Dis..

[29] Longo A.B., E Ward W. (2016). PUFAs, Bone Mineral Density, and Fragility Fracture: Findings from Human Studies. Adv. Nutr. Int. Rev. J..

[30] Hinojosa C.A., Gonzalez-Juarbe N., Rahman M., Fernandes G., Orihuela C.J., Restrepo M.I. (2020). Omega-3 fatty acids in contrast to omega-6 protect against pneumococcal pneumonia. Microb. Pathog..

[31] Menni C., Zierer J., Pallister T., Jackson M.A., Long T., Mohney R.P., Steves C.J., Spector T.D., Valdes A.M. (2017). Omega-3 Fatty Acids Correlate with Gut Microbiome Diversity and Production of N-Carbamylglutamate in Middle Aged and Elderly Women. Sci. Rep..

[32] (2021). European Committee on Antimicrobial Susceptibility Testing. Breakpoint Tables for Interpretation of MICs and Zone Diameters, version 11.0. https://www.eucast.org/.

[33] Floros S., Toskas A., Pasidi E., Vareltzis P. (2022). Bioaccessibility and Oxidative Stability of Omega-3 Fatty Acids in Supplements, Sardines and Enriched Eggs Studied Using a Static In Vitro Gastrointestinal Model. Molecules.

[34] Carlson S.J., Fallon E.M., Kalish B.T., Gura K.M., Puder M. (2013). The role of the ω-3 fatty acid DHA in the human life cycle. JPEN J. Parenter. Enter. Nutr..

[35] Johnson J.D., Nessler J.M., Horazdovsky R.D., Vang S., Thomas A.J., Marston S.B. (2017). Serum and Wound Vancomycin Levels After Intrawound Administration in Primary Total Joint Arthroplasty. J. Arthroplast..

