# Supplementary material for: Eicosapentaenoic Acid and Docosahexaenoic Acid as an Antimicrobial Agent in Orthopedics—An In Vitro Study About the Race for Surface
Source: Pathogens. 2025 Jan 10;14(1):57. doi: 10.3390/pathogens14010057 (PMC11768219; doi:10.3390/pathogens14010057)
Supplement: Supplementary file 1 [file pathogens-14-00057-s001.zip › pathogens-3303721-supplementary.pdf]

Table S1. P-values of the two-way ANOVA for the different time points and PUFAs used for osteoblast adhesion tests.

Time	DHA 1.25 mg/L	DHA 2.5 mg/L	EPA 0.15 mg/L	EPA 0.3 mg/L
1 h	0,34548272	0,64786669	0,88979229	0,91403042
6 h	0,89612343	0,07206225	0,36313261	0,68831115
24 h	0,10852724	0,60250784	0,19302339	0,29434033

Table S2. P-values of the two-way ANOVA for the different time points and PUFAs used of the colony forming unit counts of the co-culture.

Time	DHA 1.25 mg/L	DHA 2.5 mg/L	EPA 0.15 mg/L	EPA 0.3 mg/L
1 h	0,67501589	0,37645074	0,57641265	0,36499201
6 h	0,20729734	0,14926591	0,25017377	0,53640234
24 h	0,36398097	0,10654513	0,54269209	0,77399569

Table S3. P-values of the two-way ANOVA for the different time points and PUFAs used of the osteoblast cell counts of the co-culture.

Time	DHA 1.25 mg/L	DHA 2.5 mg/L	EPA 0.15 mg/L	EPA 0.3 mg/L
1 h	0,51907695	0,27401585	1	0,13300432
6 h	9,3098E-05	0,0018285	0,00047999	0,00014883
24 h	0,62243111	0,62243111	0,62243111	0,62243111

Table S4. Results of a Turkey's multi-comparison test of the osteoblast cell counts of the co-culture with the control, solvent control and DHA 1.25 mg/L.

group1	group2	meandiff	p-adj	lower	upper	reject
control	DHA 1.25	33750	0,0005	18142,2141	49357,7859	TRUE
control	solvent control	-7500	0,4091	-23107,7859	8107,7859	FALSE
DHA 1.25	solvent control	-41250	0,0001	-56857,7859	-25642,2141	TRUE

Table S5. Results of a Turkey's multi-comparison test of the osteoblast cell counts of the co-culture with the control, solvent control and DHA 2.5 mg/L.

group1	group2	meandiff	p-adj	lower	upper	reject
control	DHA 2.5	57500	0,0059	19198,1557	95801,8443	TRUE
control	solvent control	-8750	0,8036	-47051,8443	29551,8443	FALSE
DHA 2.5	solvent control	-66250	0,0024	-104551,844	-27948,1557	TRUE

Table S6. Results of a Turkey's multi-comparison test of the osteoblast cell counts of the co-culture with the control, solvent control and EPA 0.15 mg/L.

group1	group2	meandiff	p-adj	lower	upper	reject
control	EPA 0.15	43750	0,0012	20834,9448	66665,0552	TRUE
control	solvent control	-2500	0,9504	-25415,0552	20415,0552	FALSE
EPA 0.15	solvent control	-46250	0,0008	-69165,0552	-23334,9448	TRUE

 https://doi.org/10.3390/pathogens14010057

Table S7. Results of a Turkey's multi-comparison test of the osteoblast cell counts of the co-culture with the control, solvent control and EPA 0.3 mg/L.

group1	group2	meandiff	p-adj	lower	upper	reject
control	EPA 0.3	87500	0,0004	48255,7371	126744,263	TRUE
control	solvent control	-5000	0,9331	-44244,2629	34244,2629	FALSE
EPA 0.3	solvent control	-92500	0,0003	-131744,263	-53255,7371	TRUE
